# Burnout and its Influencing Factors among Primary Health Care Providers in the North East of Iran

**DOI:** 10.1371/journal.pone.0167648

**Published:** 2016-12-08

**Authors:** Mohammad Amiri, Ahmad Khosravi, Ahmad Reza Eghtesadi, Zakieh Sadeghi, Ghasem Abedi, Mansour Ranjbar, Fardin Mehrabian

**Affiliations:** 1 Department of Public Health, School of Public Health, Shahroud University of Medical Sciences, Shahroud, Iran; 2 Center for Health Related social and Behavioral Sciences Research, Shahroud University of Medical Sciences, Shahroud, Iran; 3 Department of English Language Teaching, Shahid Beheshti Campus, Farhangian University, Mashhad, Iran; 4 Analytical Chemistry, Shahroud, Iran; 5 Health Sciences Research Center, Mazandaran University of Medical Sciences, Sari, Iran; 6 Department of Public Health, Mazandaran University of Medical Sciences, Sari, Iran; 7 Department of Health Education and Promotion, School of Health, Guilan University of Medical Sciences, Rasht, Iran; TNO, NETHERLANDS

## Abstract

**Introduction:**

Burnout is a popular research topics in service providing jobs, including the health care field. This study aimed at assessing the level of job burnout and to consider the important antecedents which might be related to job burnout among primary health care providers in Iran.

**Methods:**

The participants in this applied cross-sectional study which was conducted in 2013 were 548 primary health care providers who were randomly selected from among those working in Shahroud, Sabzevar, Neishabour, Bojnord (provinces located in the north east of Iran). Maslach Burnout Inventory (MBI) was administered to the participants and the collected data were analyzed using SPSS through chi-square test and ordinal logistic regression model.

**Results:**

The burnout mean score among the participants was 54.1 ± 27.2 and the mean scores of burnout components i.e., emotional exhaustion, depersonalization and personal accomplishment were 15.5 ± 13.6, 3.7 ± 5.4 and 35.5 ± 13.5 respectively. In terms of levels of burnout, 64.2% of the participants showed low levels (n = 352), 18.4% average levels (n = 101) and 17.3% high levels (n = 95). A significant relationship was observed between burnout, job resources and interest in job (p ≤ 0.05). However, no significant relationship was observed between burnout and the place (university) of working, age, satisfaction with income, experience, gender, level of education, marital status, housing status, having a second job and place of residence (p ≥0.05).

**Conclusion:**

Lack of personal accomplishment was highly prevalent among the participating primary health care providers. Lack of career advancement and job transfer opportunities may play a role in the burnout of primary health care providers. Therefore, paying attention to this aspect may help to reduce burnout and even increase job engagement.

## Introduction

Burnout as a prolonged response to chronic stressors on the job [1, 2] is one of the most popular research topics in occupational health psychology, which has attracted attention in service and health care providing occupations since the 1970s [3–5]. Research shows burned-out employees display poor job performance and may face serious health problems over the course of time [5]. Burnout is defined as a psychological syndrome, which involves a loss of enthusiasm for work (emotional exhaustion], a sense of pessimism (depersonalization) and a reduced sense of personal accomplishment [4, 6–12]. Burnout is an increasing negative reaction to constant job stressors and is the result of inconsistency and mismatch between the job and the worker [8, 13] and is accompanied by side effects such as low mood, decrease in the feelings of competence and low commitment to work, low productivity, high absenteeism and delay, role conflict and job turnover. Burnout is also a predictor of health problems and low levels of job satisfaction [14–16].

Various factors such as the type of occupation, role conflict, work overload, management style, lack of social support, organizational changes and fierce competition, working overtime, inappropriate working conditions, a sense of organizational inefficiencies, lack of success, lack of career advancement opportunities and strict and cumbersome laws and regulations can lead to feelings of burnout [17–19].

Results of various research projects indicate that primary health providers are susceptible to burnout [1, 3, 9, 10, 20–28]. Studies usually show a negative relationship between burnout and job satisfaction, and both events are substantially influenced by organizational structure and processes [20, 29–32].

Burnout syndrome among medical professionals negatively affects the individual, the organization and the patients [33]. This syndrome, through creating a negative self-image, negative attitude toward work and lack of communication with clients during providing care, results in a sharp drop in the quality and quantity of health services [34–37] and consequently leads to dissatisfaction with health services system. Primary health care providers, as the primary contact level of the health system with community, do various tasks in the health houses and incur a lot of pressure. Moreover, limited career advancement and job transfer opportunities, and constant and continuous dealing with rural clients from different age groups make primary health care providers prone to burnout. Therefore, understanding and prevention of burnout will have a significant role in improving the quality of services [38]. This study aimed at assessing the level of burnout among primary health care providers and it attempted to consider the important antecedent factors associated with burnout among primary health care providers in the North East of Iran.

## Methods and Materials

This applied cross-sectional research was conducted in 2013 in Iran. In this study, 50% of primary health care providers (Behvarz) who were working in health houses affiliated with universities of medical sciences in Shahroud, Sabzevar, Nishabur, Bojnoord and Semnan (provinces located in the north east of Iran) were selected using the random sampling method. In Iran, the primary level of services delivery is Health Houses. Each village contains a Rural Health House, staffed by trained ‘‘Behvarz”. The Behvaz is the primary health care provider in rural area of Iran.

The methods and proposal of this study was reviewed and approved with the Ethical Review Board and Research Committee of Shahroud University of Medical Sciences (code number: 9121).

The instruments used in the study included a 20 item demographic questionnaire and the Maslach Burnout Inventory (MBI) [7, 39]. The validity and reliability of the inventory had already been confirmed in Iran [40–42]. The Cronbach alpha of the Persian version of Maslach Burnout inventory in the Sahebzadeh’study and Pardakhtchi’ study were equal to 0.86 and 0.82, respectively [41–42]. This questionnaire was designed to assess three dimensions of Burnout syndrome, Emotional exhaustion, depersonalization and reduced personal accomplishment. The questionnaire has 22 items that are answered in terms of frequency with a 7-point, fully anchored scale (ranging from 0, “never” to 6,”every day”) [7]. In our study the reliability of this questionnaire was acceptable (alpha = 0.85). the alpha chronbach for 3 dimension of burnout including of emotional exhaustion, depersonalization and reduced personal accomplishment were 0.91, 0.92 and 0.77, respectively. Maslach and Jackson [7] classified the 3 dimensions of the questionnaire according to below scaling: Emotional exhaustion (EE) scores below 19 indicate low, 19–26 are considered average and scores over 26 are classified as high EE. Depersonalization (DP) scores over 9 indicate high DP and scores between 6–9 are average. Personal accomplishment scores between 34 and 39 are considered average and lower and over this range are classified as low and high.

After explaining the goals of the study and obtaining a verbal informed consent from the participants, MBI and the demographic questionnaire were administered anonymously to the participants by trained interviewers.

The collected data were analyzed using Stata12 through chi-square and multivariate ordinal logistic regression tests. Because the outcome variable (burnout) is a ordinal variable, we used ordinal logistic regression. To consider important antecedent factors with job burnout among primary health care providers we used a multivariate model, burnout was treated as a three-level (ordered) variable and attendance factors that had a p- value <0.2 in univariate model were included in our ordinal logistic model. Likelihood Ratio test was used to compare the final model and other models. The standard error was estimated using the bootstrap method and the significance level for all tests was set 0.05.

## Results

The results of the study showed that more than 90% of primary health care providers participating in the study were married. The mean age of the participants was 35.8± 7.5. The average work experience of the participants was 13.8±8.2 years and the average rural residence was 23.7 ± 14.7 years. More than 60 percent of the participants were settling down in rural areas. Only 13.9% and 66.2% of participants were satisfied and partially satisfied with their income and 22.6% and 68.2% were satisfied and partially satisfied with their workplace facilities. Most of participants scored a high interest in their job (58.4% was much interested and 21.5% even very much). (Table 1).

**Table 1:** Demographic characteristics among primary health care providers (n = 548) in north east of Iran, 2013.

Variable		n,%
Medical University	Shahroud	106, 19.3
	Semnan	33,6
	Bojnord	239,43.6
	Sabzevar	66, 12
	Neishbour	104, 19
Gender	Male	159, 29
	Female	389, 71
Education	High school diploma and lower	450, 82.1
	Over diploma	98,17.9
Marital status	Single	45, 8.2
	Married	503, 91.8
Second job	Yes	29, 5.3
	No	519, 94.7
Satisfaction with income	Satisfied	76, 13.9
	Partially satisfied	363, 66.2
	dissatisfied	109, 19.9
Satisfaction withFacilities	Satisfied	124, 22.6
	Partially satisfied	376, 68.6
	dissatisfied	48, 8.8
Interest in the job	Very little	19, 3.5
	little	91,16.6
	Much	320, 58.4
	Very much	118, 21.5

The analysis of data collected through MBI also indicated that 35.7% of the participating primary health care providers had average to high levels of emotional exhaustion, 49.6% showed high levels of lack of personal accomplishment and 8.8% had high levels of depersonalization.

The mean score of burnout was 54.09 ± 27.23 and the mean scores of burnout components were 15.48 ± 13.58, 3.71 ± 5.36 and 35.49 ± 13.54 respectively for emotional exhaustion, depersonalization and personal accomplishment. The findings indicate that 41.8% of the participants experienced low levels of burnout (n = 229); 52.7% had symptoms of average levels of burnout (n = 289) and 5.5% suffered from high levels of burnout (n = 30).

The results of Table 2 showed that there is some relationship between components of burnout and demographic variables. Emotional exhaustion was significantly associated with age, work experiences, gender, Interest in the job and satisfaction with income. There was a significant inverse association between depersonalization with interest in the job and facilities of workplace. Finally, interest in job was associated with reduced personal accomplishment.

**Table 2:** The frequency distribution of burnout dimensions based on demographic variables and experience, workplace facilities, interest in the job, satisfaction with income and second job variables among primary health care providers (n = 548) in north east of Iran, 2013.

Variables	Burnout Dimensions								
	Emotional exhaustion			Depersonalization			Reduced personal accomplishment		
	Low n,%	Moderate n,%	High n,%	Low n,%	Moderate n,%	Highn,%	Low n,%	Moderate n,%	High n,%
Age (Year)									
Less than 30	103,72	27,18.9	13,9.1	118,82.5	13, 9.1	12, 8.4	65, 45.5	24,16.8	54, 37.8
30–50	233,60.5	73,19	79,20.5	311,80.8	40, 10.4	34, 8.8	199,51.7	59,15.3	127, 33
Over 50	16,80	1,5	3,15	16,80	2, 10	2,10	12, 60	3, 15	5, 25
P.V	P = 0.012			P = 0.991			P = 0.640		
Experience (Year)									
Less than 10	141,70.9	39,19.6	19,9.5	167,83.9	19,9.5	13,6.5	89,44.7	31,15.6	79,39.7
10–20	139,62.1	38,17	47,21	178,79.5	24,10.7	22,9.8	112,50	40,17.9	72,32.1
Over 20	72,57.6	24,19.2	29,23.2	100,80	12,9.6	13,10.4	75,60	15,12	35,28
P.V	P = 0.007			P = 0.688			P = 0.067		
Workplace facilities									
Poor	68,54.8	26,21	30,24.2	90,72.6	18,14.5	16,12.9	63,50.8	15,12.1	46,37.1
Mediocre	249,66.2	67,17.8	60,16	310,82.4	35,9.3	31,8.2	186,49.5	61,16.2	129,34.3
Good	35,72.9	8,16.7	5,10.4	45,93.8	2,4.2	1,2.1	27,56.3	10,20.8	11,22.9
P.V	P = 0.085			P = 0.022			P = 0.356		
Interest in the job									
Very little	5,26.3	5,26.3	9,47.4	11,57.9	4,21.1	4,21.1	12,63.2	4,21.1	3,15.8
little	36,39.6	18,19.8	37,40.7	65,71.4	15,16.5	11,12.1	55,60.4	16,17.6	20,22.0
much	215,67.2	64,20	41,12.8	269,84.1	28,8.8	23,7.2	163,50.9	55,17.2	102,31.9
Very much	96,81.4	14,11.9	8,6.8	100,84.7	8,6.8	10,8.5	46,39.0	11,9.3	61,51.7
P.V	P = 0.001			P = 0.014			P = 0.001		
Satisfaction with income									
Totally satisfied	63,82.9	8,10.5	5,6.6	65,85.5	6,7.9	5,6.6	42,55.3	11,14.5	23,30.3
Partially satisfied	238,65.6	69,19	56,15.4	296,81.5	37,10.2	30,8.3	179,49.3	60,16.5	124,34.2
Dissatisfied	51,46.8	24,22	34,31.2	84,77.1	12,11	13,11.9	55,50.5	15,13.8	39,35.8
P.V	P = 0.001			P = 0.621			P = 0.853		
Gender									
Male	115,72.3	25,15.7	19,11.9	129,81.1	17,10.7	13,8.2	80,50.3	19,11.9	60,37.7
Female	237,60.9	76,19.5	76,19.5	316,81.2	38,9.8	35,9	196,50.4	67,17.2	126,32.4
P.V	P = 0.031			P = 0.913			P = 0.229		
Second job									
Yes	18,62.1	6,20.7	5,17.2	25,86.2	2,6.9	2,6.9	18,62.1	2,6.9	9,31
No	334,64.4	95,18.3	90,17.3	420,80.9	53,10.2	46,8.9	258,49.1	84,16.2	177,34.1
P.V	P = 0.948			P = 0.772			P = 0.298		
Marital status									
Single	31,68.9	7,15.6	7,15.6	32,71.1	6,13.3	7,15.6	22,48.9	7,15.6	16,35.6
married	321,63.8	94,18.7	88,17.5	413,82.1	49,9.7	41,8.2	254,50.5	79,15.7	170,33.8
P.V	P = 0.789			P = 0.155			P = 0.971		
Education									
Diploma and lower	283,62.9	82,18.2	85,18.9	366,81.3	42,9.3	42,9.3	229,50.9	66,14.7	155,34.4
More than diploma	69,70.4	19,19.4	10,10.2	79,80.6	13,13.3	6,6.1	47,48	20,20.4	31,31.6
P.V	P = 0.118			P = 0.334			P = 0.365		
Total	352,64.2	101,18.4	95,17.3	445,81.2	55,10	48,8.8	276,50.4	86,15.7	186,33.9

The analysis of the variables showed a statistically significant relationship between burnout, workplace facilities and interest in the job (p≤0.05), whereas the relationships between burnout and other variables including the universities under study, age, satisfaction with income, job experience, gender, level of education, marital status, housing status, having a second job and place of residence were not significant (p≥0.05).

Finally, the variables under study were examined in a multivariate model. These variables included age, gender, work experience, satisfaction with income, interest in the job, workplace facilities and having a second job. Since the dependent variable had three layers (low, average and high levels of burnout), ordinal logistic regression model was used. Workplace facilities and interest in the job were the two factors which remained in the final model which was examined using maximum likelihood ratio (LR test). Results in Table 3 show the lack of workplace facilities can increase the level of burnout by 1.8 times. In this model, the odds of increase in burnout level decrease by 0.29, 0.27 and 0.59 times respectively among people with very high, high and low levels of interest in the job. Cut-off points of 1 and 2 in the model show the effect of independent variables on average to high levels of burnout.

**Table 3:** The relationship between burnout and physical facilities and interest in the job among primary health care providers (n = 548) studied in 2013 by multivariate analysis using ordinal logistic regression model.

		Composite Burnout	Emotional exhaustion	depersonalization	personal accomplishment
variable		OR (95%CI)	OR (95%CI)	OR (95%CI)	OR (95%CI)
Workplace facilities	no	1	1	1	1
	Yes	0.55 (0.39–0.78)	0.75 (0.51–1.09)	0.8 (0.52–1.25)	0.72 (0.51–1.01)
Interest in the job	low	1	1	1	1
	average	0.42 (0.27–0.66)	0.24 (0.15–0.37)	0.44 (0.27–0.72)	1.52 (0.99–2.34)
	high	0.46 (0.27–0.78)	0.11(0.05–0.20)	043 (0.23–0.82)	3.19 (1.90–5.35)
sex	male	--	1	--	--
	Female	--	1.69 (1.11–2.57)	--	--
education	Diploma and lower	--	1	--	--
	More than diploma	--	0.47 (0.28–0.78)	--	--
Satisfaction with income	low	--	1	--	--
	average	--	2.53 (1.31–4.89)	--	--
	high	--	5.59 (2.65–11.80)	--	--
Experience in job (year)	Less than 10	--	--	--	1
	10–20	--	--	--	0.74 (0.52–1.08)
	Over 20	--	--	--	0.59 (0.38–0.93)
Cut1		-1.86 (-2.859–0.865)	0.88 (-0.22–1.97)	0.70 (0.2–1.17)	0.04 (-0.42–0.51)
Cut2		1.443 (0.444 2.441)	2.04 (0.93–3.14)	1.60 (1.09–2.10)	0.73 (0.26–1.20)

## Discussion

According to the findings, the frequency of high emotional exhaustion in the 30–50 age group was more than that in other age groups. Level of emotional exhaustion showed a significant relationship with age, which is consistent with the findings of Iglesias and colleagues [3], Arab and colleagues [43] and Mirabzadeh and colleagues [44]. Piko [20], however, found no relationship between age and emotional exhaustion. Lizano and colleagues [45] and Garrosa and colleagues [46] also reported no significant relationship between age and emotional exhaustion. These results are not consistent either with findings of this study nor with the findings of other studies carried out in Iran [40, 47 and 48].

The results of the study also show a significant relationship between work experience and emotional exhaustion, which is consistent with the results of Iglesias and colleagues [3] and Boyas and colleagues [49]. Apparently, lack of career advancement opportunities for primary health care providers makes many of them show higher levels of emotional exhaustion as they grow older and more experienced.

As for the relationship between exhaustion and gender, the results indicated a significant relationship which is consistent with what Williams and colleagues [25] and Garrosa and colleagues reported [46]. Lizano and colleagues [45], Iglesias and colleagues [3] and Piko referred to the absence of the relationship between gender and emotional exhaustion, which does not conform to recent results [20].

No significant relationship was observed in this study between marital status and education level with emotional exhaustion, which is consistent with the results of Lizano, and colleagues [3], Piko [20] and Iglesias and colleagues [45], but inconsistent with the results of Williams and colleagues [25].

The findings of this study further indicated a significant relationship between depersonalization and workplace facilities and interest in the job; however, there was no significant relationship between depersonalization with gender, marital status, level of education, age and experience. Lizano and colleagues also found no relationship between age, gender, level of education and depersonalization, which accords with the recent results [45]. Iglesias and colleagues also reported no relationship between depersonalization and gender, age group and marital status, which is consistent with recent findings [3]. Garrosa and colleagues also pointed to the absence of the relationship between gender and depersonalization, which is in line with recent results [46]. Iglesias [3] and Boyas [49], however, found a significant relationship between work experience and depersonalization, which is inconsistent with the results of this study. Williams and colleagues also reported a significant relationship between gender and depersonalization [25], which does not accord with the recent findings. Piko also found a relationship between gender, age and education, and depersonalization, which is different from the recent results [20].

In this study no significant relationship was found between lack of personal accomplishment and gender, marital status, level of education, spouse's level of education, spouse's job, and satisfaction with income, workplace facilities, age and experience. Williams and colleagues also reported no relationship between gender, marital status and lack of personal accomplishment, which is consistent with some part of the recent results [25]. Piko also found a relationship between gender and education and lack of personal accomplishment but no relationship was found with age, which is consistent with some part of the recent findings [20]. Moreover, Iglesias and colleagues reported no relationship between lack of personal accomplishment and gender, age, place of residence and work experience but a relationship with marital status, which is partially consistent with recent results [3]. Garrosa and colleagues in their study also found no relationship between lack of personal accomplishment and gender, which is consistent with the recent results [46].

The results of the study further indicated a significant relationship between the frequency of burnout and workplace facilities and interest in the job, but no significant relationship was found between age, satisfaction with income, experience, gender, education, marital status, place of residence, housing status and having a second job, which is consistent with the findings of Arab and colleagues [43], Arefi and colleagues [50], Mirabzadeh and colleagues [44]. Chin and colleagues referred to the absence of the relationship between burnout, marital status and age groups, which is consistent with the recent results [51]. The findings of this study, however, are not in line with the results of Rashedi and colleagues [48] and Talaei [40], who reported a relationship between burnout and marital status. It seems that the intense interest in the job by primary health care providers is one of the reasons which help them to tolerate difficulties and show low job burnout.

Mean frequencies of emotional exhaustion, depersonalization and lack of personal accomplishment were 15.5±13.6, 3.7± 5.4 and 35.5± 13.5, respectively. Van Bogaret and colleagues [24] in their study reported the mean frequencies of emotional exhaustion, depersonalization and lack of personal accomplishment as 11.6± 3.8, 4.3± 1.5 and 34.6± 2.3, respectively, which is similar to the our findings.

One limitation of this study was the lack of a previous study in primary health care providers to compare pur results with. Another limitation is of a cross-sectional nature and therefore does not allow us to draw any causal relationships. Wide health houses distributions and sampling method for assessing a representative sample is a strength of this study. Assessment of psychological, environmental and social factors impacts on burnout in a longitudinal study may be considered for future research. Considering our results, longitudinal studies on emotional exhaustion and personal accomplishment dimensions and its associated factors will gain insight into.

## Conclusion

Lack of personal accomplishment was the burnout dimension which showed the highest frequency among the primary health care providers. Primary health care providers currently have no career advancement and job transfer opportunities. However, the findings of this study imply that allowing experienced primary health care providers to transfer to health houses which are closer to the city, and allowing them to continue their education and improve their academic and career status may improve the professional condition of primary health care providers.

## Supporting Information

S1 File Standard Questionnaire of Burnout and its Influencing Factors among Primary Health Care Providers in the North East of Iran. (DOCX)
