# Supplementary material for: Burnout and its Influencing Factors among Primary Health Care Providers in the North East of Iran
Source: PLoS One. 2016 Dec 8;11(12):e0167648. doi: 10.1371/journal.pone.0167648 (PMC5145173; doi:10.1371/journal.pone.0167648)
Supplement: S1 File — (DOCX) [file pone.0167648.s001.docx]

**Standard Questionnaire of Burnout and its Influencing Factors among Primary Health Care Providers**

**in the North East of Iran (The questionnaire's language is in native Persian)**

**« پرسشنامه طرح تحقیقاتی بررسی فرسودگی شغلی بهورزان و عوامل موثر بر آن در دانشگاه­های علوم پزشکی شمال شرق ایران »**

با سلام و احترام

پرسشنامه حاضر در جهت تعیین فرسودگی شغلی پرسنل طراحی شده است. حوصله و دقت شما در تکمیل این پرسشنامه موجب افزایش و صحت جمع آوری اطلاعات خواهد شد. از این رو خواهشمند است با اعطای وقت گرانقدر خود، محققین را در دستیابی به نتایج صحیح و دقیق رهنمون سازید. بدیهی است که کلیه اطلاعات ثبت شده در این پرسشنامه محرمانه تلقی خواهد شد.

**مشخصات فردی:**

1. سن به سال: ....................
2. سابقه کاری به سال: ................
3. جنسیت: مرد□ زن□
4. میزان تحصیلات: پنجم ابتدایی□ سیکل□ دیپلم□ فوق دیپلم □ لیسانس□ فوق لیسانس□
5. وضعیت تأهل: مجرد□ متأهل□ مطلقه□ همسرمرده□
6. تعداد فرزندان: .......... نفر
7. شغل همسر: .....................
8. میزان تحصیلات همسر: بیسواد □ پنجم ابتدایی□ سیکل□ دیپلم□ فوق دیپلم□ لیسانس□ فوق لیسانس□ دکتری□
9. قومیت: فارس□ ترکمن□ سیستانی□ بلوچ□ ترک□ کرد□ قزاق□ سایر□
10. محل سکونت فعلی: روستای اصلی □ روستای قمر□ روستای مجاور□ مرکز بخش□ مرکز شهرستان□
11. چند سال در این محل سکونت دارید؟ .......... سال
12. وضعیت مسکن: ملک شخصی□ اجاره یا رهن□ در فضای مسکونی خانه بهداشت□ زندگی با پدر و مادر یا فامیل و دیگران□
13. آیا خانه بهداشت شما، روستای قمر تحت پوشش دارد؟ بلی□ خیر□
14. آیا در خانه بهداشت فضای مسکونی جهت بهورز وجود دارد؟ بلی□ خیر□
15. آیا مرکز بهداشت موتور سیکلت در اختیار بهورز قرار داده است؟ بلی□ خیر□
16. وضعیت پرداخت آب، برق، گاز و تلفن خانه بهداشت چگونه است؟ دولتی□ پرداخت توسط بهورز□ یک سوم مرکز بهداشت و دو سوم بهورز□ سایر موارد□ ذکر کنید................
17. آیا غیر از شغل بهورزی به شغل دیگری اشتغال دارید؟ بلی□ خیر□
18. به چه میزان از وضعیت درآمد خود رضایت دارید؟ راضی□ نسبتاً راضی□ ناراضی□
19. وضعیت امکانات ضروری زندگی شما چگونه است؟ ضعیف□ متوسط□ خوب□
20. به چه میزان به شغل خود علاقه دارید؟ خیلی کم□ کم□ زیاد□ خیلی زیاد□

**سئوالات پرسشنامه فرسودگی شغلی**

| **عنوان** | **فراوانی** | | | | | | |
| --- | --- | --- | --- | --- | --- | --- | --- |
|  | **هرگز** | **سالی چندبار** | **ماهانه** | **ماهی چندبار** | **هر هفته** | **هفته ای چندبار** | **هر روز** |
| 1. **احساس می کنم که از نظر عاطفی از کارم خسته شده ام.** |  |  |  |  |  |  |  |
| 1. **احساس می کنم که در پایان کار روزانه از کار افتاده ام.** |  |  |  |  |  |  |  |
| 1. **صبح که از خواب بر می خیزم از اینکه مجبورم روز دیگری کار کنم احساس خستگی شدید می کنم.** |  |  |  |  |  |  |  |
| 1. **احساس می کنم سر و کار داشتن با مراجعان در تمام روز بر من فشار می آورد.** |  |  |  |  |  |  |  |
| 1. **احساس می کنم که کار مرا از پا درآورده است.** |  |  |  |  |  |  |  |
| 1. **از شغلم احساس ناکامی می کنم.** |  |  |  |  |  |  |  |
| 1. **احساس می کنم که در شغلم بی مورد سختی می کشم.** |  |  |  |  |  |  |  |
| 1. **کارکردن مستقیم با مراجعان باعث استرس برای من می شود.** |  |  |  |  |  |  |  |
| 1. **احساس می کنم به آخر خط رسیده ام.** |  |  |  |  |  |  |  |
| 1. **می توانم به خوبی احساس مراجعان را درباره مطلب مطرح شده درک کنم.** |  |  |  |  |  |  |  |
| 1. **من به شیوه بسیار مؤثری به حل مشکلات مراجعان می پردازم.** |  |  |  |  |  |  |  |
| 1. **احساس می کنم به صورت مثبتی از طریق کار خود بر زندگی مراجعان تأثیر می گذارم.** |  |  |  |  |  |  |  |
| 1. **احساس می کنم پرانرژی و فعال هستم.** |  |  |  |  |  |  |  |
| 1. **به راحتی می توانم محیط آرامی را برای مراجعان ایجاد کنم.** |  |  |  |  |  |  |  |
| 1. **پس از کار صمیمانه با مراجعانم، احساس شادمانی و نشاط می کنم.** |  |  |  |  |  |  |  |
| 1. **احساس می کنم کارهای ارزشمند زیادی را در این شغل به انجام رسانیده ام.** |  |  |  |  |  |  |  |
| 1. **در کارم مسائل عاطفی را با آرامش کامل سر و سامان می دهم.** |  |  |  |  |  |  |  |
| 1. **احساس می کنم با بعضی از مراجعانم مانند اشیاء رفتار می کنم.** |  |  |  |  |  |  |  |
| 1. **احساس می کنم از وقتی به این کار پرداخته ام، نسبت به مراجعان بی عاطفه شده ام.** |  |  |  |  |  |  |  |
| 1. **نگرانم شغلم مرا از نظر عاطفی خشن کرده باشد.** |  |  |  |  |  |  |  |
| 1. **واقعاً برایم مهم نیست که برای بعضی مراجعان چه پیش می آید.** |  |  |  |  |  |  |  |
| 1. **احساس می کنم که مراجعان مرا به خاطر بی توجهی به بعضی مشکلاتشان سرزنش می کنند.** |  |  |  |  |  |  |  |
